# 2144. Duration of perioperative antibiotic prophylaxis in ventriculoperitoneal shunt surgery in children

**DOI:** 10.1093/ofid/ofac492.1764

**Published:** 2022-12-15

**Authors:** Ayumi Tada, Satoshi Ihara, Junichi Suwa, Kimihiro Taniguchi, Meiwa Shibata, Hanako Funakoshi, Yuho Horikoshi

**Affiliations:** Division of Infectious Diseases, Department of Pediatrics, Tokyo Metropolitan Children's Medical Center, Fuchu, Tokyo, Japan; Department of Neurosurgery, Tokyo Metropolitan Children's Medical Center, Fuchu, Tokyo, Japan; Department of Pharmacy, Tokyo Metropolitan Children's Medical Center, Fuchu, Tokyo, Japan; Division of Infectious Diseases, Department of Pediatrics, Tokyo Metropolitan Children's Medical Center, Fuchu, Tokyo, Japan; Division of Infectious Diseases, Department of Pediatrics, Tokyo Metropolitan Children's Medical Center, Fuchu, Tokyo, Japan; Division of Infectious Diseases, Department of Pediatrics, Tokyo Metropolitan Children's Medical Center, Fuchu, Tokyo, Japan; Division of Infectious Diseases, Department of Pediatrics, Tokyo Metropolitan Children's Medical Center, Fuchu, Tokyo, Japan

## Abstract

**Background:**

Placement of ventriculoperitoneal (VP) shunt is an important treatment for pediatric hydrocephalus. Although prolonged perioperative antibiotic prophylaxis has theoretically no benefit in reducing device-associated infections, duration of antibiotic prophylaxis for VP shunt placement in pediatrics is not well established. As a part of antimicrobial stewardship program (ASP), our ASP team recommended to stop perioperative antibiotic prophylaxis for sterile medical placement within 48 hours following surgery in April 2017.

Our aim of this study was to evaluate rate of VP shunt-associated infections following shunt placement between children received < 48 hours and ≧ 48 hours of perioperative antibiotic prophylaxis.

**Methods:**

Children aged 15 years old or younger who underwent VP shunt insertion between April 2014 and November 2021 were enrolled at Tokyo Metropolitan Children's Medical Center. Children with co-existing infection at time of surgery were excluded. Rates of VP shunt-associated infections following 1 month and 6 months of post-surgical periods were compared between children who received < 48 hours and ≧ 48 hours of perioperative antibiotic prophylaxis.

**Results:**

A total of 110 children were identified. Among them, 11 cases with VP shunt-associated meningitis and 15 cases with other infections were excluded. Girl ratio was 44%. Median age was 4.5 months old (IQR 8-40). Numbers of children with cefazolin and vancomycin-contained regimen were 83 (98.8%) and 1 (1.2%), respectively. Numbers of children who received perioperative antibiotic prophylaxis for < 48 hours and ≧ 48 hours were 43 (51.2%) and 41 (48.8%), respectively. Incidence of VP shunt-associated infections for 1 month of post-surgical period in < 48 hours and ≧ 48 hours antibiotic prophylaxis groups were 4.65% (2/43) and 12.2% (5/41), respectively. (P=0.211)　Incidence of VP shunt-associated infections for 6 months of post-surgical period in < 48 hours and ≧ 48 hours antibiotic prophylaxis groups were 11.6% (5/43) and 12.2% (5/41), respectively. (P=0.936).

**Conclusion:**

Shorter duration of < 48 hours of perioperative antibiotic prophylaxis did not increase rates of VP shunt-associated infections among children in short and long terms.

**Disclosures:**

**All Authors**: No reported disclosures.

